# Radiomics for risk stratification in brain metastases: potential in melanoma brain metastases

**DOI:** 10.1007/s10585-026-10422-0

**Published:** 2026-08-07

**Authors:** Johanna Heugenhauser, Sabrina Herbst, Tamara Drucks, Christoph Scherfler, Tadeja Urbanic Purkart, Eva Maria Hassler, Richard Partl, Marlene Leoni, Kariem Mahdy Ali, Sarah Iglseder, Christian Freyschlag, Stephanie Mangesius, Astrid Grams, Meinhard Nevinny-Stickel, Martha Nowosielski

**Affiliations:** 1 Department of Neurology, Medical University Innsbruck, Anichstrasse 35, 6020 Innsbruck, Austria; 2 Institute of Information Systems Engineering, TU Wien, Vienna, Austria; 3 Faculty of Computer Science, Data Mining and Machine Learning, University of Vienna, Vienna, Austria; 4 Department of Neurology, Medical University Graz, Graz, Austria; 5 Department of Radiology, Medical University Graz, Graz, Austria; 6 Diagnostic Institute of Radiology, LKH Murtal, Judenburg, Austria; 7 Department of Radiation Oncology, Medical University Graz, Graz, Austria; 8 Diagnostic & Research Institute of Pathology, Medical University Graz, Graz, Austria; 9 Department of Neurosurgery, Medical University Graz, Graz, Austria; 10 Department of Neurosurgery, Medical University Innsbruck, Innsbruck, Austria; 11 Department of Radiology, Medical University Innsbruck, Innsbruck, Austria; 12 Neuroimaging Research Core Facility, Medical University Innsbruck, Innsbruck, Austria; 13 Department of Radiation Oncology, Medical University Innsbruck, Innsbruck, Austria

**Keywords:** Radiomics, Risk score, Brain metastases, Magnetic resonance imaging

## Abstract

The identification of quantitative non-invasive imaging biomarkers, including radiomics, may complement molecular characterization and thereby improve clinical management of neuro-oncological patients. We aimed to identify imaging predictors with improved performance over clinical parameters to stratify patients with brain metastases into high and low-risk groups for overall survival (OS). 422 patients recruited by two neuro-oncological centers were included with first diagnosis of brain metastases from different primary tumors. From each patient, 15 clinical parameters and a total of 321 radiomic features extracted from cerebral MRI were employed in prediction models to classify patients into low- and high-risk groups for OS. The best performing model was a bootstrap aggregating model including only clinical features (test set: macro F-1 = 0.62, accuracy = 0.72), while the combined and radiomic datasets led to poorer results (test set: macro F-1 = 0.60, accuracy = 0.67; test set: macro F-1 = 0.62, accuracy = 0.52, respectively). However, in the subgroup of melanoma patients (n = 54), the radiomic dataset showed better predictive power over clinical and combined dataset (test set: macro F-1 score = 0.71, accuracy = 0.77). 80% and 67% of melanoma patients were correctly classified into the low- and the high-risk group for OS, respectively. Clinical features remained the strongest predictors of risk across patients with brain metastases from different primary tumors. Although, in melanoma patients, radiomic features provided better prediction of the survival outcome compared to clinical parameters alone.

**Supplementary Information:**

The online version contains supplementary material available at 10.1007/s10585-026-10422-0.

## Introduction

Brain metastases (BM) are the most common brain tumors in adults with a high morbidity and mortality rate. Lung and breast cancers, as well as melanomas, renal cell and gastrointestinal tumors are among the primary tumors that most commonly metastasize to the brain [1, 2]. In previous years, treatment modalities were limited to surgical resection and radiation therapy. Fortunately, new targeted-therapies demonstrated good responses to the primary tumor with additional promising efficacy regarding some BM of specific molecular subtypes [3–6]. However, the prognosis of most patients remains poor with an overall median survival of only a few months. 

For more accurate patient-specific prognosis prediction, stratification scores e.g. the “recursive partitioning analysis (RPA) score” or the “graded prognostic assessment (GPA) score” were developed. These scores are based on clinical parameters such as Karnofsky performance status (KPS), age, presence of extracranial metastases, systemic disease status, and number of BM [3, 7, 8]. At present, the number of BM remains the only radiologic parameter used for prognostic assessment, emphasizing the need for additional imaging-based biomarkers to enhance prognostic accuracy.

Diffusion-weighted imaging (DWI) is a widely available MRI sequence and an essential component of brain tumor assessment [9]. It exploits the Brownian motion of water molecules to generate image contrast, enabling quantification of the apparent diffusion coefficient (ADC). In general, highly cellular tumor regions show more restricted diffusion and therefore lower ADC values. In BM, mean ADC has been associated with survival, and ADC changes at the tumor edge may indicate a more locally aggressive phenotype [10, 11]. However, these MRI-derived parameters represent only a fraction of the quantitative information embedded in medical imaging.

Radiomics refers to the extraction of high-dimensional quantitative imaging features from defined volumes of interest to characterize tissue properties in a reproducible manner. As tumors are highly heterogeneous at the phenotypic, physiologic, and genomic levels, radiomics may provide additional insights into tumor biology through comprehensive texture analysis of the entire lesion [12]. Previous studies demonstrated promising results for radiomics in improving survival prediction and risk stratification in patients with BM and glioblastoma [13–18]. In line with this, our group previously showed that texture analysis of ADC maps improved established clinical risk models in patients with BM [19].

Building on prior findings, this study aims to evaluate radiomic features extracted from ADC maps and post-contrast T1-weighted images in patients with BM. By integrating clinical, molecular, and radiomic data, we will apply machine learning models to enable stratification of patients into high- and low-risk groups for overall survival.

## Methods

### Patients and clinical data

This was a multicenter prospective and retrospective study at the Medical University of Innsbruck and the Medical University of Graz. Adult patients with a first diagnosis of BM from various primary tumors such as lung, breast, melanoma, gastrointestinal, and renal cell were prospectively included between 2020 and 2022 at the Medical University of Innsbruck, and retrospectively over a 20-year period (2000–2023) at the Medical University of Graz and the Medical University of Innsbruck. The retrospective and prospective data analysis was approved by the local ethics committee of the Medical University of Innsbruck and Medical University of Graz, in addition to a written informed consent that was gathered from all patients included prospectively. From each patient, 15 clinical features (age, sex, Karnofsky performance status, systemic disease status, presence of extracranial metastases, number of BM, primary tumor and available individual prognostic molecular status e.g., HER2, BRAF) were obtained by chart review (Suppl. Table 1). Patients with a solitary or up to three BM, a histological diagnosis of the primary tumor, and sufficient MRI data (T1-weighted imaging, with and without contrast (CE-T1w, T1w); FLAIR or T2-weighted (T2w) imaging and DWI including ADC maps) were included. BM of a volume of ≤ 1 cm^3^ were excluded as the ADC maps analysis could be affected by partial volume effects [20].

### Magnet resonance imaging acquisition

Owing to the multicenter design of the trial, multiple MRI systems were used, all operating at a 1.5 T or 3 T field strength. Patient distributions according to MRI field strength and acquisition center are summarized in Suppl. Table 2. A 3D T1w imaging protocol including magnetization-prepared rapid acquisition with gradient echo (MP-RAGE) MRI sequences or T1w spin-echo (SE) images with contrast enhancement (0.1 mmol/kg gadopentetate dimeglumine) was performed at each center, resulting in images of similar resolution, with a slice thickness of at least 1.5 mm. All patients underwent axial FLAIR or T2w imaging with a slice thickness of at least 2 mm. Diffusion weighting was applied with b-values at 0 and 1000 s/mm^2^ in all patients.

### Postprocessing of MRI data

**Fig. 1:**
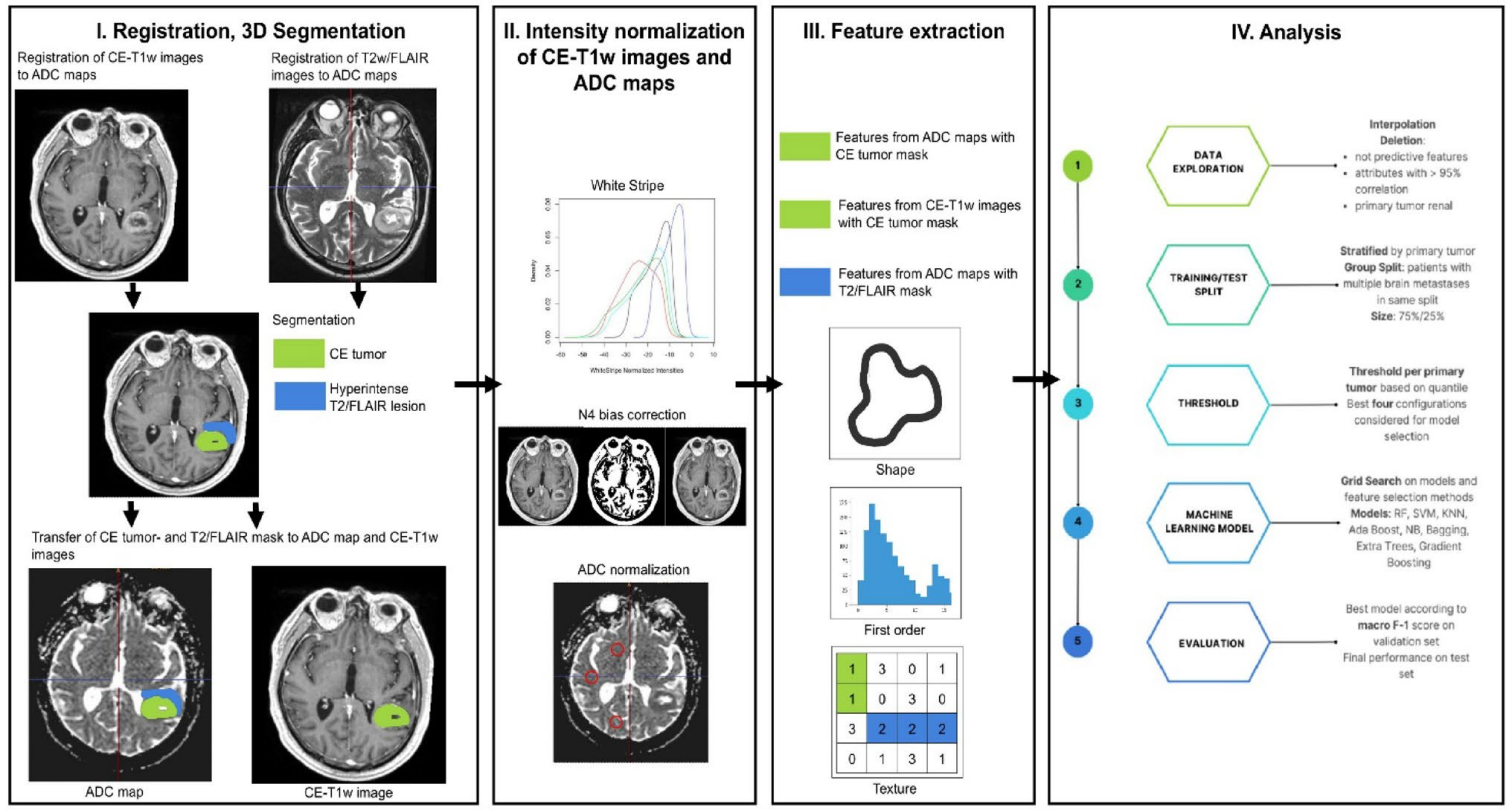
Workflow I: pseudo anonymized imaging and clinical data will be summarized. Afterwards, images (CE-T1w and T2/FLAIR images) will be co-registered to ADC maps. CE tumor volumes as well as hyperintense T2/FLAIR tumor volumes will be segmented. These segmentations are further used as regions of interest (ROIs) and will be mapped to the corresponding ADC maps. The CE tumor volumes (CE tumor mask) will also be mapped to CE-T1w images. II: As we are analyzing CE-T1w images from different scanners and institutions images need to undergo image normalization (White stripe normalization and N4 bias correction). In addition, ADC maps are normalized. III: Radiomic features will be extracted from the ADC maps (CE tumor mask and T2/FLAIR mask) and CE-T1w images (CE tumor mask). IV: Radiomics together with clinical features will be used to build different machine learning models to establish a new risk model. Abbreviations: CE: contrast enhancing, T1w: T1-weighted, T2w: T2-weighted, FLAIR: Fluid-Attenuated Inversion Recovery, 3D: 3-dimensional, ADC: Apparent diffusion coefficient, RF: random forest, SVM: support vector machine, KNN: k-nearest neighbors, NB: Naïve Bayes

Image postprocessing was performed in reference to a method already published by Kickingereder et al. [21] (Fig. 1). First, all MR images were registered to the ADC maps by using the linear image registration tool with a mutual information algorithm and a 6-degree of freedom transformation from the Functional Magnetic Resonance Imaging of the Brain (FMRIB) software library (FSL-FLIRT [22–26]). Image intensity normalization (using the N4 Bias Correction [27, 28] and WhiteStripe [29, 30] packages implemented in Python v.3) was performed on CE-T1w sequences to transform arbitrary MR signal intensities into standardized intensity ranges, to generate well-defined inputs for quantitative radiomic feature extraction. In order to normalize ADC, we used a method published by Hagiwara et al. [31]. To reduce variability among ADC from different scanners, a patient-specific relative ADC (rADC) was calculated by the mean value of normal-appearing white matter in the contralateral hemisphere (detailed method is given in Suppl. Material).

### Segmentation and volumetry on MRI

Contrast-enhanced (CE) tumor lesions in CE-T1w images (excluding dura, blood vessels, and/or central necrotic area), and hyperintense T2/FLAIR lesions (excluding the contrast enhanced and necrotic tumor portions) were segmented using a semi-automated active contour method (ITK-SNAP 3.4.0 [32]). This segmentation tool demonstrated excellent reliability and high efficiency in 3D segmentation and has already been used in different projects for tumor volumetry in our group [32, 33]. All segmentations were quality checked by a neurologist with 6 years of experience in neuroimaging (J.H.).

### Radiomic feature extraction

Radiomics feature extraction was performed using the open-source package PyRadiomics (version 3.0.1) in Python (https://pyradiomics.readthedocs.io/en/latest/) [34]. Images were resampled to an in-plane voxel spacing of 1 × 1 mm while preserving the original slice thickness. Gray-level discretization was performed using a fixed bin width of 0.1. Radiomic features were extracted from (A) the CE tumor mask on ADC maps, (B) the hyperintense T2/FLAIR lesions mask on ADC maps and (C) the CE tumor mask on CE-T1w images (Fig. 1). For patients with multiple BM, feature values were extracted from each lesion, and the mean value across all lesions was used for further analysis. Extracted radiomic features included 14 shape, 18 first order, and 75 s order features subdivided to gray level co-occurrence matrix (GLCM), gray level dependence matrix (GLDM), gray level run length matrix (GLRLM), gray level size zone matrix (GLSZM), and neighboring gray tone difference matrix (NGTDM). In total 321 radiomic features were extracted. In the Suppl. Table 1, all extracted radiomic features are summarized.

For radiomic features, feature selection was performed using Pearson correlation analysis to identify and remove redundant variables. Pairwise correlations among all extracted features were calculated, and features with a high correlation coefficient (r > 0.95) were considered redundant. In each highly correlated pair, one feature was excluded from subsequent statistical analyses.

### Risk group definition

To stratify patients into low- and high-risk groups, we applied the supervised principal components regression framework proposed by Bair and Tibshirani [35]. Because survival outcomes strongly depend on the underlying primary tumor entity, thresholds were determined separately for each primary tumor type. Candidate thresholds were evaluated using survival time quantiles ranging from 0.4 to 0.7. Patients with observed survival times below the selected threshold were assigned to the high-risk group, whereas patients surviving beyond the threshold were assigned to the low-risk group.

As the dataset contained censored survival data, threshold determination was based only on patients with observed death events. For censored patients, Kaplan–Meier survival estimates were used to estimate the probability of survival beyond the selected threshold time point, thereby allowing probabilistic assignment despite incomplete follow-up data [35]. Patients with renal cancer were excluded due to the low sample size (n = 9).

### Model training and statistical analysis

Data were split into a training (75%) and test (25%) set stratified by primary tumor type. The test set was held out throughout model development and was used exclusively for final performance evaluation. All preprocessing and model development steps, including feature selection, random forest feature importance ranking, survival threshold optimization, and hyperparameter tuning, were performed exclusively within the training dataset.

To optimize the survival-based risk stratification and machine learning models, the training dataset was further divided into a secondary training set (75%) and a validation set (25%). In addition, a custom cross-validation split was implemented for hyperparameter optimization. The validation set was used for model selection and hyperparameter optimization, while the independent test set remained untouched until the final evaluation.

Using a gradient boosting classifier, models were trained for all 65 possible combinations of survival cutoffs (six quantiles across five primary tumor entities). Accuracy and macro-evaluated F-1 score were calculated for each combination, and only the four best-performing models according to the macro F-1 score were retained for further analysis. The macro F-1 score was chosen to avoid biased splits with highly imbalanced class distributions that could artificially inflate accuracy while providing limited predictive performance. To identify the optimal machine learning model and hyperparameter configuration, a comprehensive grid search across multiple classifiers was performed. A detailed overview of all evaluated models and hyperparameters is provided in Suppl. Table 3. Next, we used a random forest to calculate feature importances and trained the models using only the 10, 20, and 30 most important features, which was performed exclusively within the training data.

The final model and hyperparameter configuration selected on the basis of validation performance were subsequently evaluated on the independent test set. With the same best-performing model subgroup analyses stratified by primary tumor were subsequently conducted to reassess performance.

Results of OS are reported as median with 95% confidence intervals. P-values less than 0.05 were considered significant. Statistical analyses were performed using R software version 4.2.1. Machine learning computations were performed with Python version 3.9.7.

## Results

### Patient characteristics

In total, 422 patients (220 female, 202 male) with a total of 519 BM were included in this study. 102 patients were recruited retrospective at the Medical University of Graz and 320 patients (32 prospective and 288 retrospective) at the Medical University of Innsbruck. 340 patients had a singular BM, while 67 patients had two and 15 patients had three BM. 207 patients were suffering from lung cancer (small and non-small cell lung cancer), 54 patients from melanoma, 40 from breast cancer, 37 from gastrointestinal cancer, 9 from renal cell carcinoma, and 75 from other primary tumor (e.g., prostate cancer, bladder cancer). In Table 1 clinical and demographic information is displayed.

**Table 1:** Clinical and demographic information of the study population

	Primary tumor						
	Total	Lung	Melanoma	Breast	Gastrointestinal	Renal Cell	Others
Number of patients, n (%)	422 (100.0)	207 (49.0)	54 (12.8)	40 (9.5)	37 (8.8)	9 (2.1)	75 (17.8)
*Sex*							
Female, n (%)	220 (52.1)	103 (49.8)	23 (42.6)	40 (100.0)	12 (32.4)	2 (22.2)	40 (53.3)
Male, n (%)	202 (47.9)	104 (50.2)	31 (57.4)	0 (0.0)	25 (67.6)	7 (77.8)	35 (46.7)
Median age at diagnosis, years (95% CI)	63 (62–64)	64 (62–65)	64 (61–68)	55 (50–60)	65 (62–68)	66 (58–74)	60 (57–64)
*Number of patients with*							
1 BM, n (%)	340 (80.5)	164 (79.2)	44 (81.4)	31 (77.5)	31 (83.8)	8 (88.9)	62 (82.7)
2 BM, n (%)	67 (15.9)	37 (17.9)	5 (9.3)	8 (20.0)	5 (13.5)	1 (11.1)	11 (14.7)
3 BM, n (%)	15 (3.6)	6 (2.9)	5 (9.3)	1 (2.5)	1 (2.7)	0 (0.0)	2 (2.6)
*Location*							
Supratentorial, n (%)	312 (73.9)	151 (72.9)	49 (90.7)	25 (62.5)	26 (70.2)	8 (88.9)	53 (70.7)
Infratentorial, n (%)	63 (14.9)	27 (13.0)	4 (7.4)	10 (25.0)	8 (21.6)	1 (11.1)	13 (17.3)
Both, n (%)	47 (11.2)	29 (14.1)	1 (1.9)	5 (12.5)	3 (8.1)	0 (0.0)	9 (12.0)
*KPS at diagnosis*							
< 70, n (%)	98 (23.2)	41 (19.8)	16 (29.6)	9 (22.5)	14 (37.8)	2 (22.2)	16 (21.3)
> 70, n (%)	324 (76.8)	166 (80.2)	38 (70.4)	31 (77.5)	23 (62.2)	7 (77.8)	59 (78.7)
*RPA stage at diagnosis*							
I, n (%)	79 (18.7)	42 (20.3)	3 (5.6)	11 (27.5)	4 (10.8)	2 (22.2)	18 (24.0)
II, n (%)	251 (59.6)	127 (61.3)	37 (68.5)	21 (52.5)	18 (48.6)	6 (66.7)	42 (56.0)
III, n (%)	91 (21.7)	38 (18.4)	14 (25.9)	8 (20.0)	15 (40.6)	1 (11.1)	15 (20.0)
*GPA score at diagnosis*							
0–1.0, n (%)	–	26 (12.6)	7 (13.0)	10 (25.0)	16 (43.3)	2 (22.2)	–
1.5–2.5, n (%)	–	120 (58.0)	16 (29.6)	8 (20.0)	9 (24.3)	1 (11.01)	–
3.0, n (%)	–	47 (22.7)	19 (35.2)	16 (40.0)	12 (32.4)	4 (44.5)	–
3.5–4.0, n (%)	–	14 (6.7)	12 (22.2)	6 (15.0)	0 (0.0)	2 (22.2)	–
Neurosurgery, n (%)	170 (40.3)	81 (39.1)	17 (31.5)	17 (42.5)	17 (45.9)	2 (22.2)	36 (48.0)
*Radiotherapy*							
WBRT, n (%)	211 (50.0)	105 (50.7)	24 (44.4)	22 (55.0)	19 (51.4)	3 (33.3)	38 (50.7)
SRT, n (%)	170 (40.3)	87 (42.0)	25 (46.3)	23 (57.5)	12 (32.4)	1 (11.1)	22 (29.3)
Median survival, months (95% CI)	19 (17–22)	19 (16–23)	24 (17–31)	26 (16–35)	9 (6–11)	27 (0–58)	17 (12–22)
*Survival at end of study*							
Death, n (%)	249 (59.0)	125 (60.4)	31 (57.4)	18 (45.0)	28 (75.7)	5 (55.6)	42 (56.0)
Alive, n (%)	88 (20.9)	46 (22.2)	14 (25.9)	13 (32.5)	1 (2.7)	1 (11.1)	13 (17.3)
Unknown, n (%)	85 (20.1)	36 (17.4)	9 (16.7)	9 (22.5)	8 (21.6)	3 (33.3)	20 (26.7)

KPS: Karnofsky performance score, RPA: recursive partitioning analysis score, GPA: graded prognostic assessment, BM: brain metastases, WBRT: whole brain radiotherapy, SRT: stereotactic radiotherapy, n: number, CI: confidence interval

### Best performing models for risk stratification

**Fig. 2:**
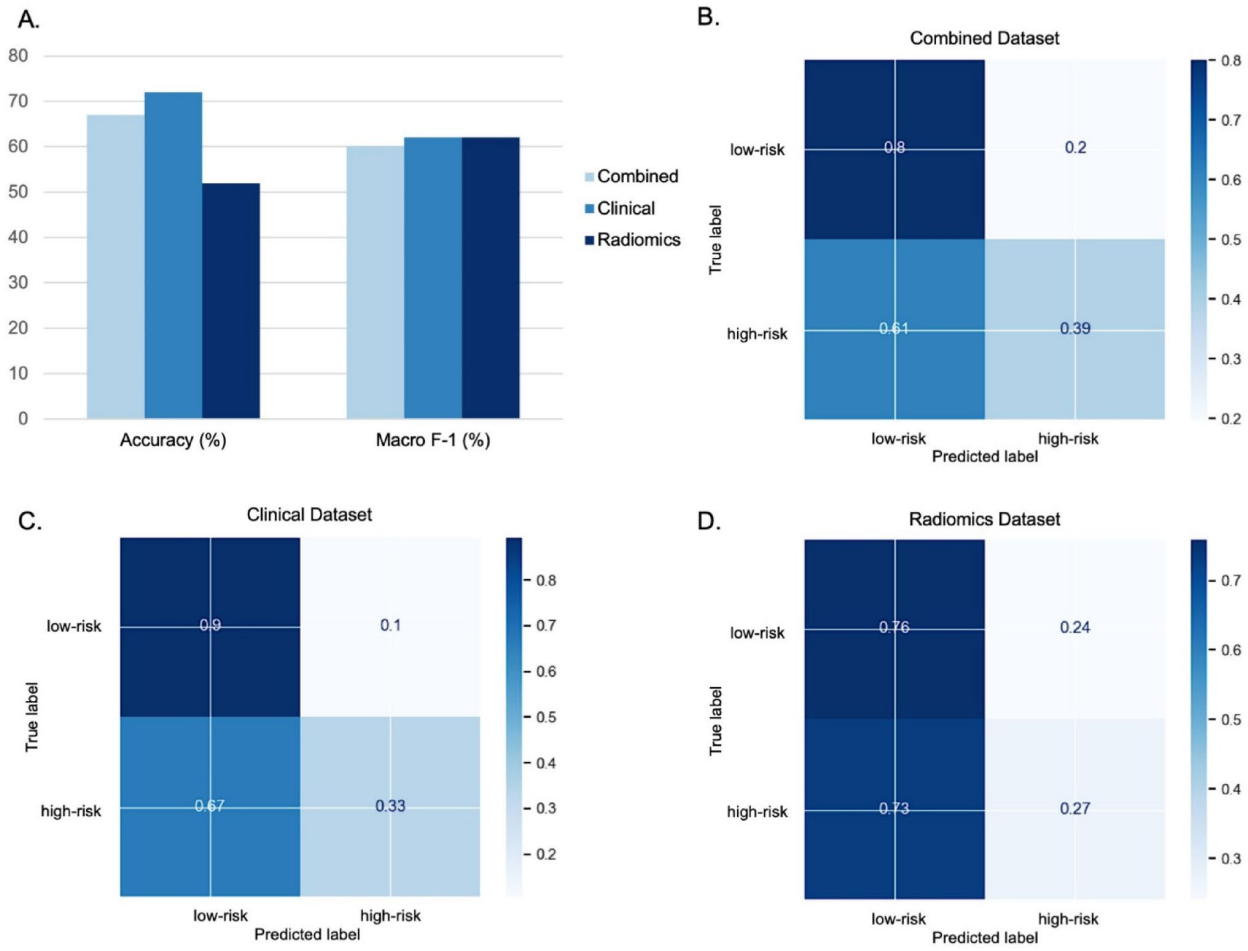
The best performing models with different datasets. The highest accuracy and macro F-1 score was seen in the bootstrap aggregating model trained on the clinical dataset **A**. The best performing models trained on the combined (bootstrap aggregating model) **B**, clinical (bootstrap aggregating model) **C** and radiomics dataset (gradient boosting model) **D**, correctly classified a high percentage of low-risk patients. In contrast, only a low percentage of high-risk patients were classified correct

All 422 patients were allocated in a 3:1 ratio to a training (n = 317) and test (n = 105) set. With a bootstrap aggregating model, containing 10 most important features (2 clinical and 8 radiomic features, Table 2) we stratified patients into a high- and low-risk group for OS (test set: macro F-1 = 0.60, accuracy = 0.67, Fig. 2A). With this model, 80% of low-risk patients but only 39% of high-risk patients were correctly classified (Fig. 2B). The overall best-performing model was a bootstrap aggregating model using clinical features only (10 best-performing clinical features, Table 2): test set: macro F-1 = 0.62, accuracy = 0.72, Fig. 2A). The gradient boosting model using radiomic features only (20 best-performing radiomic features, Table 2) performed the worst: test set: macro F-1 = 0.62, accuracy = 0.52 (Fig. 2A). With the best-performing models trained on the clinical, and radiomics datasets 90% and 76% of low-risk patients were correctly classified. However, they misclassified 67%, and 73% of high-risk patients, respectively (Fig. 2C and D).

**Table 2:** Most important features used in individual machine learning models

Machine learning model		
Combined dataset	Clincal dataset	Radiomics dataset
KPS	Age at diagnosis of primary tumor	GLSZM gray level variance (CE tumor mask on CE-T1w)
first order maximum (CE tumor mask on CE-T1w)	Age at diagnosis of BM	first order kurtosis (CE tumor mask on ADC)
GLCM cluster prominence (CE tumor mask on CE-T1w)	KPS	GLSZM zone entropy (CE tumor mask on CE-T1w)
GLCM contrast (CE tumor mask on CE-T1w)	Gender	GLCM cluster shade (CE tumor mask on ADC)
GLCM id (CE tumor mask on CE-T1w)	Number of BM	first order range (CE tumor mask on ADC)
age at diagnosis of BM	Molecular marker	first order interquartile range (CE tumor mask on CE-T1w)
GLDM small dependence high gray level emphasis (CE tumor mask on CE-T1w)	Systemic disease status	first order maximum (CE tumor mask on CE-T1w)
first order 10 percentile (CE tumor mask on ADC)	Presence of extracranial metastases	GLSZM high gray level zone emphasis (CE tumor mask on CE-T1w)
first order range (CE tumor mask on CE-T1w)	Primary tumor	first order mean (T2/FLAIR mask on ADC)
GLCM difference variance (CE tumor mask on CE-T1w)	Location of BM	GLCM idn (CE tumor mask on CE-T1w)
		GLCM cluster prominence (CE tumor mask on CE-T1w)
		GLCM cluster shade (T2/FLAIR mask on ADC)
		first order maximum (CE tumor mask on ADC)
		GLCM difference variance (CE tumor mask on ADC)
		GLDM small dependence high gray level emphasis (CE tumor mask on CE-T1w)
		GLSZM gray level non uniformity normalized (CE tumor mask on CE-T1w)
		GLRLM long run low gray level emphasis (CE tumor mask on CE-T1w)
		GLCM difference variance (CE tumor mask on CE-T1w)
		GLCM cluster prominence (CE tumor mask on ADC)
		shape elongation (CE tumor mask on CE-T1w)

T1w: T1-weighted, BM: brain metastasis, KPS: Karnofsky performance score, CE: contrast-enhancing, GLCM: gray level cooccurrence matrix, id: inverse difference, idn: inverse difference normalized, GLDM: gray level cooccurrence matrix, GLRLM: gray level run lenght matrix, GLSZM: gray level size zone matrix

### Prediction of high- and low-risk in melanoma patients

**Fig. 3:**
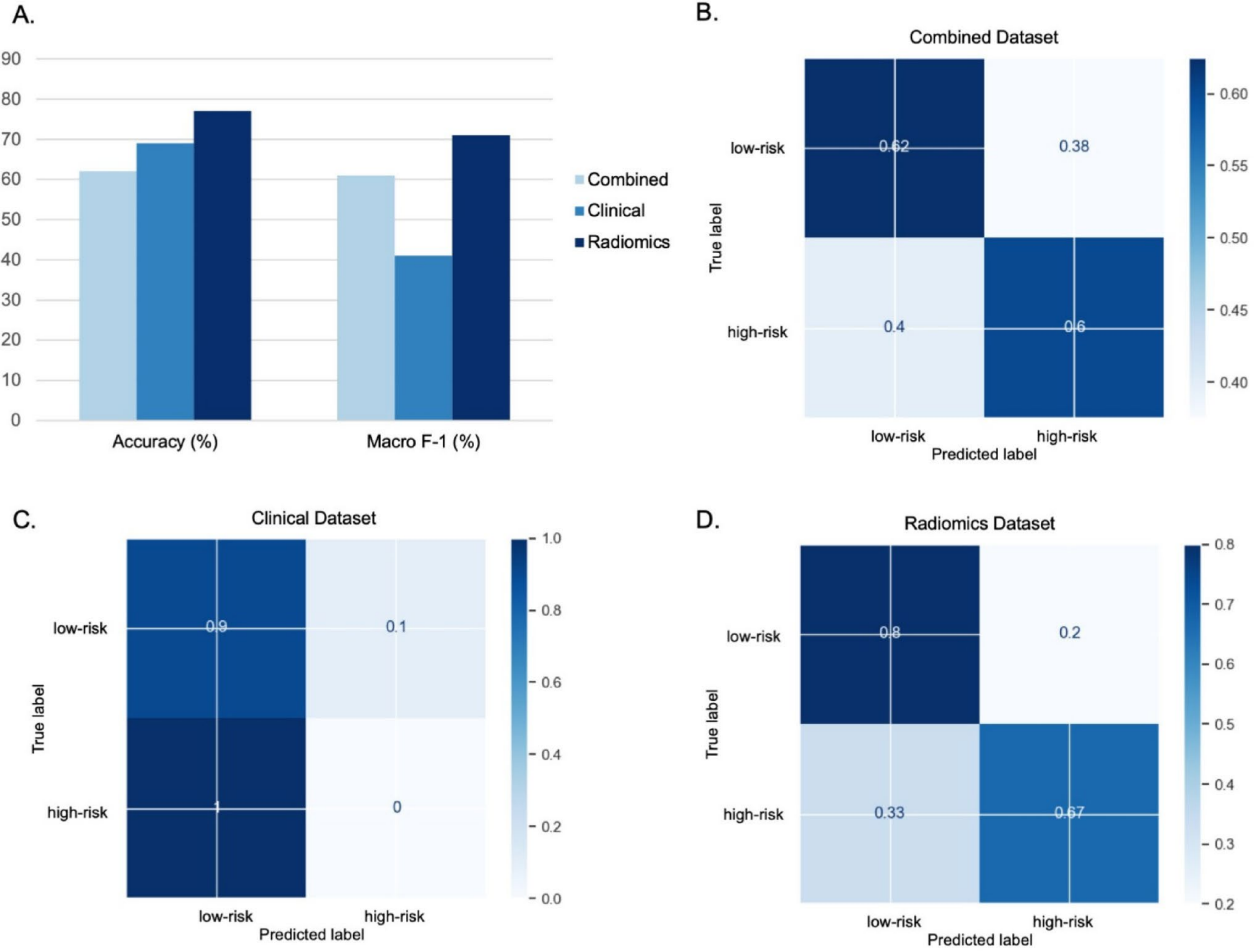
The best performing models with different datasets in melanoma patients with brain metastases. The highest accuracy and macro F-1 score was seen in the gradient boosting model trained on the radiomics dataset **A**. The best performing models trained on the combined **B**, clinical **C** and radiomics dataset **D** in the subgroup of melanoma patients, classified a high percentage of low-risk patients correct. The gradient boosting model trained on the radiomics dataset classified the highest percentage of high-risk patients correct

Applying the same machine learning models and best-performing features from the overall cohort (Table 2), the melanoma subgroup showed significantly better predictive performance using the radiomic dataset (test set: macro F-1 score = 0.71, accuracy = 0.77), compared to the clinical dataset (test set: macro F-1 score = 0.41, accuracy = 0.69) and the combined dataset (test set: macro F-1 score = 0.60, accuracy = 0.62) (Fig. 3A). The gradient boosting model using 20 radiomic features was able to predict 80% of low-risk patients and 67% of high-risk patients correctly (Fig. 3D).

For the remaining subgroups confusion matrices are provided in Suppl. Figures 1–4, and results for the different datasets (combined, clinical, and radiomics) are summarized in Suppl. Table 4.

## Discussion

Comprehensive non-invasive characterization of brain tumors on MRI has recently emerged as a promising field of research. This approach aims to utilize the full potential of medical imaging data and allows non-invasive, three-dimensional, and quantitative characterization of neoplastic tissue [12, 36, 37]. The ultimate goal of this technique is the identification of quantitative imaging biomarkers that may complement molecular and clinical characterization and thereby improve clinical management of neuro-oncological patients [17, 18, 21, 38, 39]. Recent studies have applied radiomics to predict local control and outcome after stereotactic radiosurgery, identify the primary tumor origin, and predict molecular markers in patients with BM [36, 37, 40–43]. However, the prognostic value of radiomics for survival prediction in BM remains insufficiently explored, while current risk stratification models still rely primarily on clinical and molecular parameters.

In our study, we stratified 422 patients with BM originating from different primary tumors. We extracted 321 radiomic features and 15 clinical variables and trained multiple machine learning models using a combined dataset as well as separate clinical and radiomics datasets to classify patients into high- and low-risk groups for overall survival (OS). Our results demonstrated that clinical features alone provided the strongest prediction of survival outcome in patients with BM, underscoring the continued importance of established clinical prognostic parameters for patient stratification. In the overall cohort, radiomic features did not provide substantial additional predictive value beyond clinical information. Model performance was primarily driven by the accurate identification of low-risk patients, likely reflecting a bias toward the majority class and a tendency to classify a large proportion of patients as low-risk. Consequently, high-risk patients may have been misclassified, potentially limiting clinical applicability. Importantly, the clinical model used in the present study was based mainly on readily available baseline parameters, including age, KPS, systemic disease status, presence of extracranial metastases, and the number of BM. Recent studies have shown that additional clinical and treatment-related factors, such as immune checkpoint inhibitor therapy and serum lactate dehydrogenase levels, may provide substantial prognostic information [44]. Therefore, comprehensive clinical models and easily accessible clinical markers should remain a central component of risk stratification strategies before considering more labor-intensive radiomic approaches.

Interestingly, in the subgroup of 54 melanoma patients, the predictive power of radiomic features alone was superior compared to clinical features or all features combined. When applying solely radiomic features, our machine learning model correctly classified 80% of low-risk patients and 67% of high-risk patients. This suggests that radiomic features derived from melanoma BM may have greater predictive potential than those obtained from BM of other primary tumor entities. Melanoma BM may represent a particularly informative radiomic phenotype, as imaging appears to capture biologically relevant tumor characteristics beyond conventional morphologic assessment. Supporting this concept, Meißner et al. previously demonstrated that intracranial BRAF V600 mutation status could be predicted noninvasively using radiomic features, underscoring the potential of radiomics to reflect clinically and molecularly meaningful information in melanoma BM [36]. Consistent with these findings, multiple studies in extracranial metastatic melanoma have reported that CT-based radiomic signatures provide prognostic information beyond established clinical parameters and may improve risk stratification, further supporting the biological relevance of radiomic phenotypes in melanoma [45–50]. Melanin, the biomolecule responsible for pigmentation and frequently present in melanoma BM, may induce susceptibility effects through paramagnetic metal scavenging, resulting in T1w hyperintensity. Susceptibility-weighted MRI is highly sensitive to these artifacts, which radiomic analysis may capture more comprehensively than conventional visual assessment [51, 52]. Radiomic features derived from CE-T1w imaging in our melanoma subcohort, including first-order intensity metrics (e.g., maximum, mean) and texture-based features (e.g., GLSZM and GLCM-derived measures), may indirectly capture melanin-associated signal characteristics such as intrinsic T1w hyperintensity. This may partly explain the improved predictive performance of radiomics in melanoma BM observed in our cohort, although these features remain non-specific and likely reflect broader tissue heterogeneity. Overall, our findings suggest a tumor-specific role of radiomics in BM, with the most promising results observed in melanoma. In contrast, predictive performance remained limited for other tumor entities, including lung cancer despite the larger sample size.

### Limitations

Several limitations should be considered when interpreting our findings. First, the observed superiority of radiomics in melanoma BM was based on a relatively small subgroup and therefore requires cautious interpretation and external validation. In addition, radiomics rely on a complex and time-intensive workflow, including image normalization, segmentation, and high-dimensional feature extraction, which may limit its immediate clinical applicability compared to easily accessible clinical prognostic markers. Furthermore, the long study period and multicenter design likely introduced heterogeneity in treatment strategies and MRI acquisition protocols, potentially affecting the reproducibility of extracted radiomic features, particularly ADC-derived parameters. Although N4 bias field correction, White Stripe normalization, and ADC normalization were applied prior to feature extraction, no dedicated radiomic feature harmonization technique was performed, and residual scanner- and center-related variability cannot be excluded. This may partly explain why ADC-based radiomic features did not improve survival prediction in our cohort despite previously reported prognostic value [19]. Finally, the models were developed and tested only on internal datasets without external validation, which may limit their generalizability. In summary, these factors likely contribute to the limited performance and generalizability of our models. Our results should therefore be interpreted as a proof-of-concept rather than definitive evidence of clinical utility. The observed variability between tumor types suggests that the predictive value of radiomics in BM may be highly tumor-specific and warrants further investigation in larger, more balanced cohorts.

### Future perspectives

Beyond MRI radiomics, several emerging technologies are expected to further advance personalized neuro-oncology. Molecular imaging techniques such as PET may provide complementary biological information [53, 54], while digital health technologies, including Internet of Things (IoT)-based platforms [55], may facilitate longitudinal patient monitoring and personalized care. In addition, three-dimensional (3D) printing has shown promise for surgical planning, education, and patient-specific treatment visualization [56]. Although these approaches were beyond the scope of the present study, their integration with MRI-based radiomics represents an interesting direction for future research.

## Conclusion

Clinical features alone provided the strongest prediction of survival outcome across the overall cohort. While the predictive value of radiomics varied substantially between primary tumor types, these findings suggest a tumor-specific role of radiomic biomarkers in BM. Interestingly, our findings suggest that melanoma BM may exhibit distinct imaging patterns, and that radiomic features derived from these patterns may improve survival prediction beyond established clinical risk models. Future studies should focus on validating these findings in larger multicenter cohorts and on developing integrative prognostic models combining clinical and imaging biomarkers.

## Supplementary Information

Below is the link to the electronic supplementary material.

Supplementary file1 (DOCX 657 kb)

## Data Availability

The data will be made available upon reasonable request.

## Declarations

### Originality and presentations

The authors confirm the originality of this study.
