# Supplementary material for: Radiomics for risk stratification in brain metastases: potential in melanoma brain metastases
Source: Clin Exp Metastasis. 2026 Aug 7;43(4):39. doi: 10.1007/s10585-026-10422-0 (PMC13451392; doi:10.1007/s10585-026-10422-0)
Supplement: Supplementary file 1 [file 10585_2026_10422_MOESM1_ESM.docx]

**Radiomics for Risk Stratification in Brain Metastases: Potential in Melanoma Brain Metastases**

# Supplementary Material

# 1. Methods

## 1.1 Normalization of ADC maps

To reduce variability among ADC from different scanners, a patient-specific relative ADC (rADC) was calculated, with a method published by Hagiwara et al. [1]. In each patient, three spherical volumes of interest (VOI) (5 mm diameter) were drawn in the anterior, middle and posterior area around 3 mm above the upper end of the lateral ventricles in the contralateral hemisphere in which the metastasis was located. Each VOI was treated as a single region of interest with a specific normal-appearing white matter value to calculate the mean normal-appearing white matter value from all three VOIs. By dividing each ADC map by the corresponding mean normal-appearing white matter value the rADC map was calculated.

# 2. Table and Figures

Supplementary Table 1 All Clinical and Radiomic Features

| Clinical Features | Radiomic Features | |
| --- | --- | --- |
| gender | shape | least axis length |
| age at diagnosis of primary tumor |  | major axis length |
| age at diagnosis of BM |  | maximum 2D diameter column |
| primary tumor |  | maximum 2D diameter row |
| molecular marker |  | maximum 2D diameter slice |
| number of BM |  | maximum 3D diameter |
| location of BM |  | mesh volume |
| presence of extracranial metastases |  | minor axis length |
| systemic disease status |  | sphericity |
| therapy of primary tumor |  | surface area |
| chemotherapy |  | surface volume ratio |
| neurosurgery |  | elongation |
| WBRT |  | flatness |
| SRT |  | voxel volume |
| KPS | first order | 10 percentile |
|  |  | 90 percentile |
|  |  | energy |
|  |  | entropy |
|  |  | interquartile range |
|  |  | kurtosis |
|  |  | maximum |
|  |  | mean absolute deviation |
|  |  | mean |
|  |  | median |
|  |  | minimum |
|  |  | range |
|  |  | robust mean absolute deviation |
|  |  | root mean squared |
|  |  | skewness |
|  |  | total energy |
|  |  | uniformity |
|  |  | variance |
|  | GLCM | autocorrelation |
|  |  | cluster prominence |
|  |  | cluster shade |
|  |  | cluster tendency |
|  |  | contrast |
|  |  | correlation |
|  |  | difference average |
|  |  | difference entropy |
|  |  | difference variance |
|  |  | id |
|  |  | idm |
|  |  | idn |
|  |  | idmn |
|  |  | imc 1 |
|  |  | imc 2 |
|  |  | inverse variance |
|  |  | joint average |
|  |  | joint energy |
|  |  | joint entropy |
|  |  | MCC |
|  |  | maximum probability |
|  |  | sum average |
|  |  | sum entropy |
|  |  | sum squares |
|  | GLDM | dependence entropy |
|  |  | dependence non uniformity |
|  |  | dependence non uniformity normalized |
|  |  | dependence variance |
|  |  | gray level non uniformity |
|  |  | gray level variance |
|  |  | high gray level emphasis |
|  |  | large dependence emphasis |
|  |  | large dependence high gray level emphasis |
|  |  | large dependence low gray level emphasis |
|  |  | low gray level emphasis |
|  |  | small dependence emphasis |
|  |  | small dependence high gray level emphasis |
|  |  | small dependence low gray level emphasis |
|  | GLRLM | gray level non uniformity |
|  |  | gray level non uniformity normalized |
|  |  | gray level variance |
|  |  | high gray level run emphasis |
|  |  | long run emphasis |
|  |  | long run high gray level emphasis |
|  |  | long run low gray level emphasis |
|  |  | low gray level run emphasis |
|  |  | run entropy |
|  |  | run length non uniformity |
|  |  | run length non uniformity normalized |
|  |  | run percentage |
|  |  | run variance |
|  |  | short run emphasis |
|  |  | short run high gray level emphasis |
|  |  | short run low gray level emphasis |
|  | GLSZM | gray level non uniformity |
|  |  | gray level non uniformity normalized |
|  |  | gray level variance |
|  |  | high gray level zone emphasis |
|  |  | large area emphasis |
|  |  | large area high gray level emphasis |
|  |  | large area low gray level emphasis |
|  |  | low gray level zone emphasis |
|  |  | size zone non uniformity |
|  |  | size zone non uniformity normalized |
|  |  | small area emphasis |
|  |  | small area high gray level emphasis |
|  |  | small area low gray level emphasis |
|  |  | zone entropy |
|  |  | zone percentage |
|  |  | zone variance |
|  | NGTDM | busyness |
|  |  | coarseness |
|  |  | complexity |
|  |  | contrast |
|  |  | strength |

BM: brain metastasis, WBRT: whole brain radiotherapy, SRT: stereotactic radiotherapy, KPS: Karnofsky performance score, 2D: 2-dimensional, 3D: 3-dimensional, GLCM: gray level cooccurrence matrix, id: inverse difference, idm: inverse difference moment, idn: inverse difference normalized, idmn: inverse difference moment normalized, imc: informational measure of correlation, MCC: maximal correlation coefficient, GLDM: gray level cooccurrence matrix, GLRLM: gray level run length matrix, GLSZM: gray level size zone matrix, NGTDM: neighboring gray tone difference matrix

Supplementary Table 2 MRI field strengths across acquisition centers

| Center |  | Innsbruck prospective | Innsbruck retrospective | Graz retrospective |
| --- | --- | --- | --- | --- |
| Fieldstrength | 1.5 T | 12 | 203 | 61 |
|  | 3 T | 20 | 85 | 41 |

Supplementary Table 3 Models and Hyperparameters

| Models | Hyperparameters |
| --- | --- |
| Random Forest | n estimators: [250, 300, 350] |
| Random Forest | max features: [sqrt, log2] |
| Random Forest | max depth: [3, 5, 7, None] |
| Random Forest | criterion: [gini, entropy] |
| Support Vector Machine | kernel: [rbf, poly] |
| Support Vector Machine | C: [1, 10, 100, 1000] |
| Support Vector Machine | gamma: [0.1, 1, 10] |
| Support Vector Machine | degree: [2, 3, 4] |
| K-Nearest Neighbors | n neighbors: [1, 5, 10, 15, 20] |
| Ada Boost | n estimators: [25, 50, 75, 100] |
| Ada Boost | learning rate: [1.0, 0.9, 0.8, 0.7] |
| Gaussian Naive Bayes |  |
| Bootstrap Aggregating | n estimators: [10, 20, 30] |
| Bootstrap Aggregating | max samples: [0.8, 0.7, 0.6] |
| Bootstrap Aggregating | max features: [1, 0.75, 0.5] |
| Extra Trees | n estimators: [75, 100, 125] |
| Extra Trees | criterion: [gini, entropy, log loss] |
| Extra Trees | min samples split: [2, 4, 6] |
| Extra Trees | max features: [log2, sqrt] |
| Gradient Boosting | n estimators: [75, 100, 125] |
| Gradient Boosting | criterion: [friedman mse, squared error] |
| Gradient Boosting | learning rate: [0.1, 0.2, 0.3] |
| Gradient Boosting | max depth: [None, 3, 4, 5] |
| Histogram Gradient Boosting | loss: [log loss, binary crossentropy] |
| Histogram Gradient Boosting | learning rate: [0.1, 0.2, 0.3] |
| Histogram Gradient Boosting | max depth: [None, 3, 5, 7, 9] |

Supplementary Table 4 Results for the different datasets (combined, clinical and radiomics) for each subgroup

|  | Combined dataset | | Clinical dataset | | Radiomics dataset | |
| --- | --- | --- | --- | --- | --- | --- |
| Primary Tumor | Accuracy | Macro F-1 | Accuracy | Macro F-1 | Accuracy | Macro F-1 |
| Lung | 0.667 | 0.556 | 0.667 | 0.597 | 0.583 | 0.515 |
| Breast | 0.778 | 0.438 | - | - | 0.778 | 0.438 |
| Melanoma | 0.615 | 0.606 | 0.692 | 0.409 | 0.769 | 0.707 |
| Gastrointestinal | 0.750 | 0.733 | 0.375 | 0.365 | 0.375 | 0.273 |
| Other | 0.632 | 0.490 | 0.895 | 0.802 | 0.684 | 0.406 |

Supplementary Fig. 1 Confusion matrices of lung cancer patients with BM

Supplementary Fig. 2 Confusion matrices of breast cancer patients with BM

Supplementary Fig. 3 Confusion matrices of gastrointestinal cancer patients with BM

Supplementary Figure 4 Confusion matrices of other cancer patients with BM

# 3. References

1.	Hagiwara A, Oughourlian TC, Cho NS, et al (2022) Diffusion MRI is an early biomarker of overall survival benefit in IDH wild-type recurrent glioblastoma treated with immune checkpoint inhibitors. Neuro Oncol 24:1020–1028. https://doi.org/10.1093/neuonc/noab276
